# 

**Published:** 1864-03

**Authors:** 


### DENTAL REGISTER

### OF THE WEST.

EDITED BY

J. TAFT AND GEO. WATT.

MARCH.—1864.

MONTHLY:
Three Dollars per Annum, in advance.

CINCINNATI:
J. TAFT, PUBLISHER.

